# Usefulness of Combined Advanced Dynamic Contrast-Enhanced and Diffusion-Weighted MRI Over Ultrasonography in Differentiating Cancer From Benign Lesions in Dense Breasts

**DOI:** 10.7759/cureus.69634

**Published:** 2024-09-18

**Authors:** Divya Muthuvel, Sudipta Mohakud, Nerbadyswari Deep, Dillip Muduly, Pankaj Kumar, Pritinanda Mishra, Suprava Naik

**Affiliations:** 1 Radiodiagnosis, All India Institute of Medical Sciences, Bhubaneswar, Bhubaneswar, IND; 2 Surgical Oncology, All India Institute of Medical Sciences, Bhubaneswar, Bhubaneswar, IND; 3 General Surgery, All India Institute of Medical Sciences, Bhubaneswar, Bhubaneswar, IND; 4 Pathology, All India Institute of Medical Sciences, Bhubaneswar, Bhubaneswar, IND

**Keywords:** breast cancer, breast neoplasms, breast ultrasonography, diffusion magnetic resonance imaging, multiparametric magnetic resonance imaging

## Abstract

Aim

To evaluate the role of dynamic contrast-enhanced magnetic resonance imaging (DCE-MRI) and diffusion-weighted imaging (DWI) in characterizing suspicious lesions in dense breasts compared to ultrasonography (USG).

Materials and methods

Eighty-two consecutive female patients with suspicious lesions in dense breast parenchyma showing the American College of Radiology Breast Imaging Reporting And Data System (ACR BI-RADS) c/d composition on mammography underwent USG, where 63 lesions of 63 patients were suspicious. They underwent multiparametric MRI, followed by histopathological evaluation (HPE) of the lesions. Statistical analysis was done to calculate the sensitivity, specificity, and accuracy of USG and MRI in lesion characterization and the combined accuracy of DCE-MRI with DWI. The receiver operating characteristic (ROC) curve analysis provided the cut-off for the apparent diffusion coefficient (ADC) value.

Results

The sensitivity, specificity, and accuracy of USG were 91.7%, 63%, and 79.4%, respectively. Kinetic curve analysis on DCE-MRI showed a type I curve only in benign lesions. Malignant lesions predominantly showed a type III curve. The sensitivity, specificity, and accuracy of DCE-MRI were 95.8%, 78.5%, and 85.7%, respectively. The optimum cut-off ADC value was 1.05x10-3 mm^2^/s with sensitivity, specificity, and accuracy of 83.3 % each. The specificity and accuracy of combined DCE-MRI with DWI were 94.4% and 88.1%, respectively.

Conclusion

Advanced MRI, including a combination of DCE-MRI kinetics and DWI, would be more effective and accurate for lesion characterization in dense breasts and act as a superior problem-solving tool compared to USG in differentiating carcinoma from benign lesions.

## Introduction

Breast cancer is the most common malignancy diagnosed in women globally [1,2]. In the year 2020, 2.3 million cases were diagnosed, and it is the leading cause of cancer death in women, with 685,000 deaths worldwide [3]. There is an increasing incidence of breast cancer in Asian countries, including India, predominantly in younger age groups. In 2018, 162,468 new breast cancer cases were detected, representing 27.7% of all new cancers among Indian women. In India, a higher proportion of patients with breast cancer are seen in the premenopausal age group between 40 and 50 years. Young breasts are often denser, reducing the sensitivity of mammography by obscuring lesions [4,5]. Women with dense breasts, i.e., a composition of the American College of Radiology Breast Imaging Reporting And Data System (ACR BI-RADS) c or d, also have an increased risk of developing breast cancer [4].

Ultrasonography (USG) can be used as a supplementary tool to mammography for effective lesion characterization. USG differentiation of lesions has low sensitivity and variable accuracy using morphologic criteria described in the BI-RADS lexicon [6,7]. Magnetic resonance imaging (MRI) more accurately describes biologically active cancers. Advanced imaging sequences such as diffusion-weighted imaging (DWI) and dynamic contrast-enhanced MRI (DCE-MRI) improve the ability to differentiate lesions [8].

The addition of USG or MRI increases the sensitivity and specificity along with a reduction in false-positive rates [5,9]. Because of its higher sensitivity, the use of breast MRI as a screening tool in patients at high risk for breast cancer and its utility in evaluating the extent of disease in patients with newly diagnosed cancer has been well established. Nevertheless, there is inadequate research on breast MRI to support its role as a problem-solving tool for inconclusive clinical or mammographic findings compared to much cheaper, quicker, and widely available USG [10]. To our knowledge, we found one retrospective study comparing the diagnostic accuracy of 3 Tesla MRI with USG in dense breasts by Pediconi et al. [10]. No other similar study from India could be found in the literature search. Thus, we tried to conduct a prospective study to compare MRI and USG to characterize suspicious lesions in dense breast parenchyma in Indian women. The secondary objective was to detect the sensitivity and specificity of individual sequences such as DWI and DCE-MRI in differentiating benign and malignant lesions.

## Materials and methods

**Figure 1:**
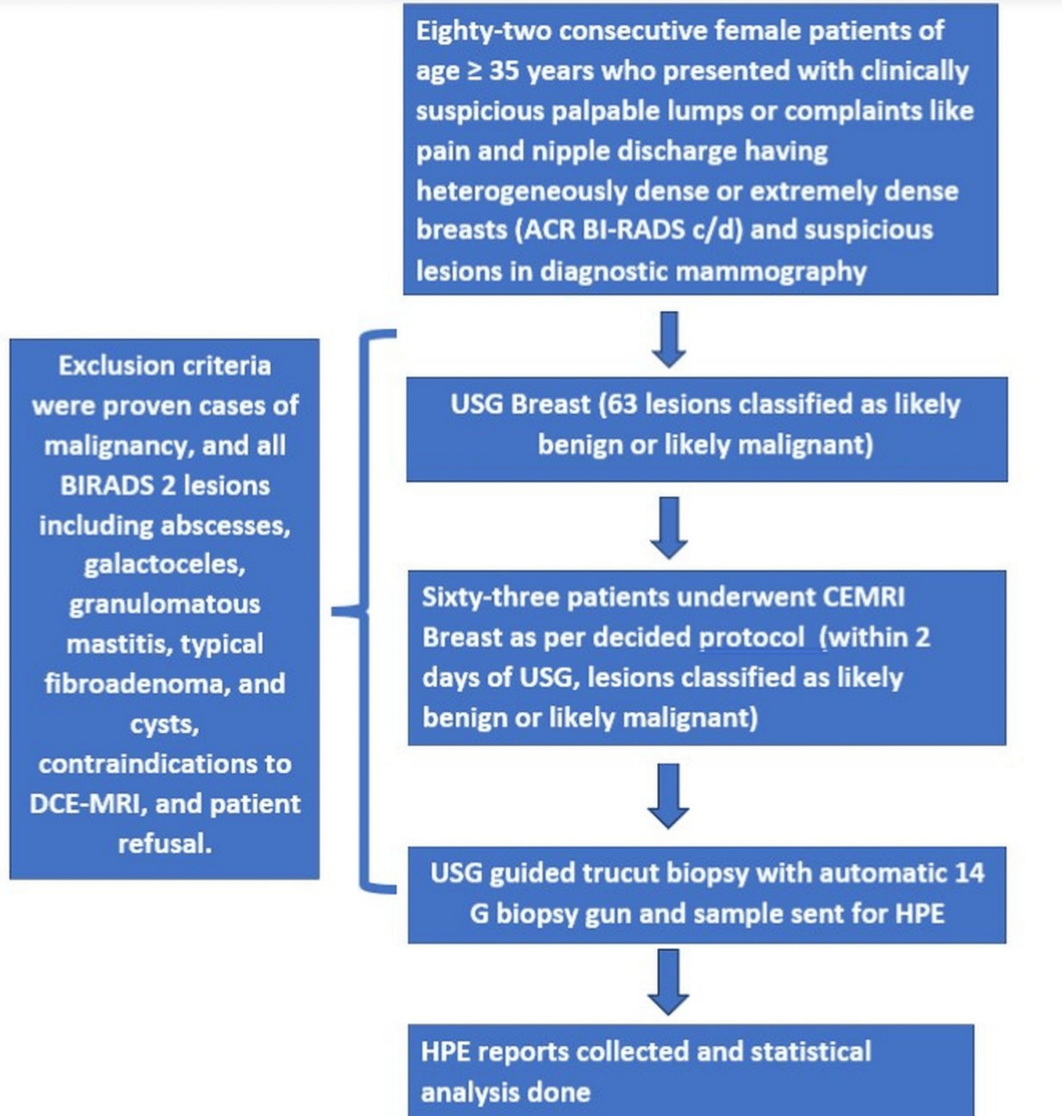
CONSORT image depicting the detailed patient selection and methodology. ACR BI-RADS: American College of Radiology Breast Imaging Reporting and Data System; USG: Ultrasonography; DCE-MRI: Dynamic Contrast-Enhanced Magnetic Resonance Imaging; HPE: Histopathological Examination; CONSORT: Consolidated Standards of Reporting Trials

The study was designed as a prospective observational study conducted in the Department of Radiology of a tertiary care teaching hospital in eastern India after obtaining clearance from the Institute Ethics Committee, spanning two years (July 2020-June 2022). Eighty-two consecutive female patients of age ≥ 35 years who presented with clinically suspicious palpable lumps or complaints such as pain and nipple discharge having heterogeneously dense or extremely dense breasts (ACR BI-RADS c/d) in diagnostic mammography with suspicious lesions were enrolled for assessment by USG. Informed consent was obtained from patients meeting the inclusion criteria. Exclusion criteria were proven cases of malignancy, and all BIRADS 2 lesions including abscesses, galactoceles, granulomatous mastitis, typical fibroadenoma, and cysts, contraindications to DCE-MRI, and patient refusal. First, the patients underwent USG by an experienced radiologist (> six years of experience in sonomammography). The USG examinations involved bilateral breasts and axillae, using a Philips iU22TM (Netherlands) USG machine with a linear probe of 5-12 MHz frequency in B mode. Color Doppler and tissue-harmonic imaging were used to characterize the lesion better. We considered the BIRADS lexicon to assess the lesions and classified them as “likely benign” (BIRADS 3 lesions) and “likely malignant” (BIRADS 4 and 5 lesions) for statistical analysis taking reference from a study by Sravani et al. [11]. The definitely benign lesions (BIRADS 2) were excluded. Sixty-three lesions of 63 patients were classified as "likely benign" and "likely malignant" lesions on USG, followed by bilateral breast MRI maximum within the next two days. The detailed patient selection and methodology are depicted in a consort diagram (Figure 1).

A normal serum creatinine (normal range=0.7-1.4 mg/dL) test was a prerequisite for MRI contrast injection. The MR mammography was performed in a 3 Tesla MRI machine (Discovery™ MR750, GE) with the patient in a prone position using a dedicated 16-array breast coil following a predetermined multiparametric protocol (Table 1).

**Table 1:** Breast MRI protocol of our study outlining the technical specifications.

SEQUENCES	TECHNICAL PARAMETERS	
T1 weighted imaging (3D axial T1 SPGR)	TR (Repetition Time)-4.8, FOV (Field of View)-35X35, slice thickness-1.6 mm, bandwidth-62.5 and matrix-350X350	
T2 weighted imaging (T2 axial FSE IDEAL)	TR-4269-5980 ms, TE (Time to Echo)-102 ms, FOV-39X39, slice thickness-5 mm, bandwidth 62.50, NEX (Number of Excitations)-1, and matrix -384X256	
T2 weighted fat suppressed imaging (T2 axial FSE ASPIR)	TR-9475-10500 ms, TE-102 ms, FOV-35X35, Slice thickness-4 mm, Bandwidth-41.67, NEX-2 and matrix-320X320	
DWI imaging with b=0 and b=800 s/mm^2^	TR-5368-9206 ms, TE-70-90 ms, FOV-35, Bandwidth-250 and NEX-12 and matrix size- 192x192	
Dynamic contrast T1 fat suppressed imaging (3D axial VIBRANT mph, pre- and post-contrast sequences)	FOV-39x39, Bandwidth-62.5, Flip angle-12, Slice thickness -4mm, matrix size-350x350. In all patients, a bolus of intravenous contrast medium gadobenate dimeglumine (Multihance^TM^, GE, USA) was administered by a pressure injector at a rate of 3 ml/sec at a dose of 0.1 mmol/kg body weight followed by 20 mL of saline infusion at the same rate. Seven consecutive phases were taken at an interval of 90 seconds	

The MRI study interpretation was done by a radiologist with more than six years of experience in breast imaging. The MRI image reviewer was blinded to USG findings.

Image analysis

In the USG study, the morphological features of the lesion were described as per ACR guidelines of BI-RADS US-lexicon, and lesions were divided into "likely benign" and "likely malignant" categories. Any lesion that was not “definitely benign” on USG (BIRADS 3 or above) underwent an MRI.

In the MRI study, conventional sequences T1- and T2-weighted imaging were primarily used to assess breast density and lesion localization. A T2 fat-suppressed sequence was used as a supportive sequence, along with a DCE-MRI, to assess the lesion morphologically.

DCE-MRI images were assessed visually, and background parenchymal enhancement was graded as a minimal, mild, moderate, and marked enhancement. The images were looked for the presence or absence of any mass or non-mass enhancement. Then, the shape, margin, or distribution (in case of non-mass) and the enhancement pattern were noted in the first post-contrast image after 90 seconds of administration of the contrast agent. The kinetic curve was assessed by placing a region of interest (ROI) at the area of maximum enhancement. The lesion was considered benign when a rising curve (type I) was noted and a plateau (type II) or washout curve (type III) was considered malignant.

DW images were assessed both qualitatively and quantitatively. Qualitative assessment was done by observing the signal pattern on high b value DWI and the corresponding ADC (apparent diffusion coefficient) map. The quantitative assessment was done by obtaining the ADC value on the ADC map. The ADC map was plotted using DWI with b-values of 0 s/mm^2^ and 800 s/mm^2^. Manual ROI was drawn on the visually assessed most restricted area (high signal intensity on DWI and corresponding low intensity in ADC map), and ADC value was determined. A cut-off value of 1.2x10^-3 ^mm^2^/s was considered to differentiate benign from malignant breast lesions.

The combined analysis of DCE-MRI with DWI was considered positive when both DCE-MRI and DWI showed positive findings. The final diagnoses were obtained from the histopathological examination (HPE) of the trucut biopsies. Sixty-three patients with 63 lesions were subjected to an ultrasound-guided core needle biopsy with a 14-gauge automatic biopsy gun after the MRI study. HPEs of these patients were compared with the results of USG and MRI.

The data collected were entered using Microsoft Excel (Microsoft® Corp., Redmond, WA). Data analysis was done with the help of Statistical Product and Service Solutions (SPSS, version 20; IBM SPSS Statistics for Windows, Armonk, NY) and MS Excel. Qualitative data were presented with the help of frequency and percentages. A p-value of less than 0.05 was considered significant. Receiver operative characteristics (ROC) analysis was used to calculate the quantitative data. The sensitivity, specificity, accuracy, and positive and negative predictive values of USG, DCE-MRI, and DWI were calculated individually, and combined DCE-MRI with DWI results were also compiled.

## Results

We included 63 patients with suspicious lesions in dense breasts (ACR BI-RADS c/d composition on mammography); the age range was between 35 and 59 years (mean age 42 years). Most patients (N=51, 81%) had type C breast composition on mammographic evaluation, followed by type D (N=12, 19%).

On HPE, there were 27 benign lesions (42.9%) and 36 malignant lesions (57.1%). The most common benign lesion was fibroadenoma (14/27 lesions), while the most common malignant one was invasive ductal carcinoma (30/36 lesions) (Table 2).

**Table 2:** The frequency distribution of the various histopathological types of breast lesions.

Histopathology Examination (HPE)	Frequency (N=63)
Benign	27
Fibroadenoma	14
Fibrocystic disease	4
Fibroproliferative disease	2
Phyllodes tumor	1
Benign features	6
Malignant	36
Invasive ductal carcinoma	30
Invasive lobular carcinoma	2
Ductal carcinoma in situ	3
Malignant phyllodes/primary sarcoma	1

On USG, 20 lesions were likely benign, 17 of which were benign, and three were malignant on HPE. Forty-three lesions were likely malignant, 10 of which were benign, and 33 were malignant (Table 3).

**Table 3:** Classification of breast lesions on ultrasonography and their histopathology examination results.

Histopathology (HPE)	Ultrasonography (USG)		
	Likely Benign	Likely Malignant	Total (n=63)
Benign	17	10	27
Malignant	3	33	36

**Figure 2:**
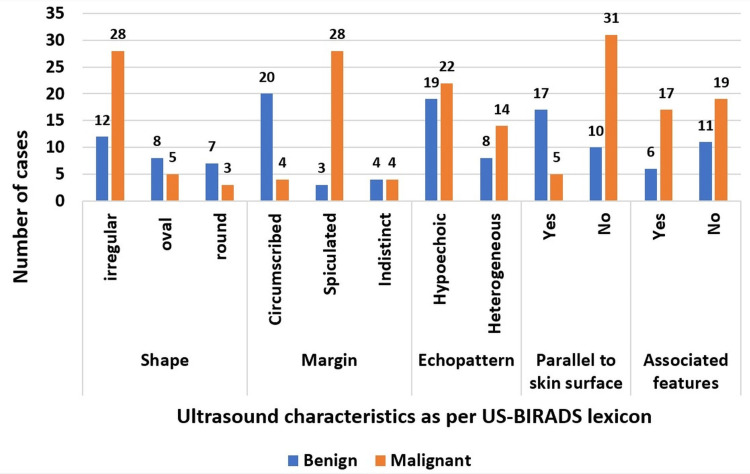
A clustered column showing the distribution of USG descriptors of BI-RADS in benign and malignant lesions.

The lesions were described with the US-BI-RADS lexicon (Figure 2), where most of the benign lesions in our study showed 12 (45.8%) cases with irregular shapes, followed by eight (29.1%) oval and seven (25%) round shapes. Seventy-five percent (N=20) of the lesions showed circumscribed margins: four cases had indistinct margins and three had spiculated margins. Nineteen (70.8%) lesions showed a hypoechoic echo pattern, and 17 lesions (62.5%) appeared parallel to the skin surface and did not show posterior acoustic features or calcifications. Only three lesions had posterior acoustic features, and one lesion showed calcific focus. Associated features such as intramammary and axillary lymphadenopathy were noted in six benign cases.

Malignant lesions predominantly showed an irregular shape and spiculated margin (N=28, 77.8% in each category). Five lesions were oval, and three lesions had a round shape. Circumscribed margins were observed in 11.1% (N=4) of the lesions. Four lesions had indistinct margins. Most lesions were not parallel to the skin surface (86.6%, N=31). Lesions were predominantly hypoechoic (60%, N=22), and the remaining appeared heterogeneous (40%, N=14) in echotexture. Posterior features and calcifications were seen only in 13.4% (N=5) and 10% (N=4) of the cases, respectively. Associated features such as skin and duct changes were observed in 46.6% (N=17) of the lesions. On correlating USG with HPE results, a P value<0.001 was derived.

Vascularity was noted in five benign and 27 malignant lesions on color Doppler. The rest of the lesions were nonvascular. The P value <0.001 was obtained, which is statistically significant.

On MRI, the amount of fibroglandular tissue was heterogeneous (type C) in 88.8 % (N=32) of cases and extreme (type D) in 11.2% (N=4). Our study showed mild background parenchymal enhancement in 56 cases (88.8%), and the rest showed either minimal (three cases) or moderate (four cases) background parenchymal enhancement. A P value of 0.6 was found with no significant association with lesion characterization.

On MRI, 18 lesions were likely benign, out of which 16 turned out to be benign, and two turned out to be malignant on HPE. On MRI 45 lesions were likely malignant, out of which 34 turned out to be malignant, and 11 turned out to be benign on HPE (Table 4).

**Table 4:** Classification of breast lesions on multiparametric MRI using MRI-BIRADS classification (considering BIRADS 3 lesions "likely benign" and BIRADS 4 and 5 lesions "likely malignant") and their histopathology results.

Histopathology	Multiparametric magnetic resonance imaging (Using MRI-BIRADS classification)		
	Likely Benign	Likely Malignant	Total (n=63)
Benign	20	7	27
Malignant	2	34	36

**Figure 3:**
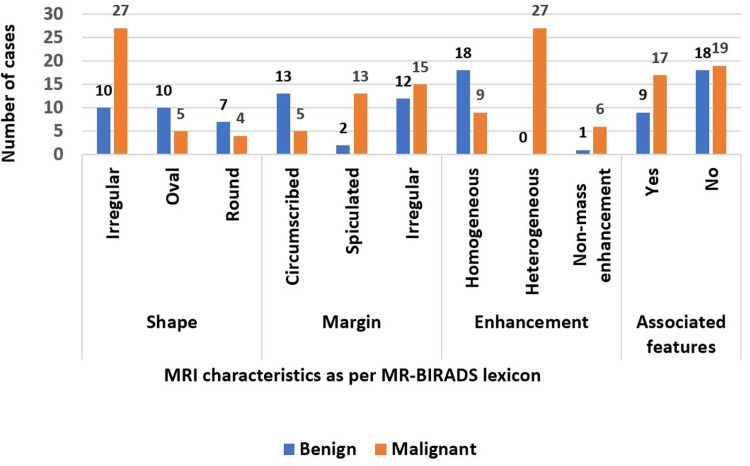
Clustered bar chart showing the distribution of MRI descriptors of BI-RADS in benign and malignant lesions.

The lesions were described as per the MR BI-RADS lexicon (Figure 3), where most of the benign lesions showed irregular and oval shapes (37.5% each, N=10 for each category), and 25% (N=7) had round shapes. Margins were circumscribed (48%, N=13) and irregular (45.8%, N=12) for each category), with two cases showing spiculated margins. A homogenous enhancement pattern (66.7%, N=18) was seen predominantly. A non-mass enhancement within the dilated retroareolar ducts was seen in one case. Another case had no enhancement characteristics. Associated lymphadenopathy was seen in 33.3% (N=9). Malignant lesions predominantly showed irregular shapes (75%, N=27) and margins (50%, N=18). Five lesions were oval, and four were round. Spiculated margins were noted in 36.1% (N=13), and the rest, 13.9% (N=5), had circumscribed margins. Most lesions (75%, N=27) had heterogeneous enhancement, and the remaining showed homogenous enhancement. Lymphadenopathy was associated with 47.2% (N=17) of the malignant lesions. Only six cases had associated skin changes.

On correlating MRI with HPE results, a P value<0.001 was derived. Type II and type III kinetic curves were seen in 13.8% (N=5) and 86.2% (N=31) of malignant lesions, respectively. Benign lesions showed type I (33.33%, N=9) and type II (66.67%, N=18) kinetic curves. We found that the sensitivity, specificity, positive predictive value (PPV), negative predictive value (NPV), and accuracy of USG were 91.7%, 63%, 76.7%, 85%, and 79.4%, respectively. DCE-MRI, along with kinetic curve analysis, yielded a sensitivity of 95.8%, specificity of 78.5%, PPV of 82.1%, NPV of 85.7%, and accuracy of 85.7% at a 95% confidence interval.

**Figure 4:**
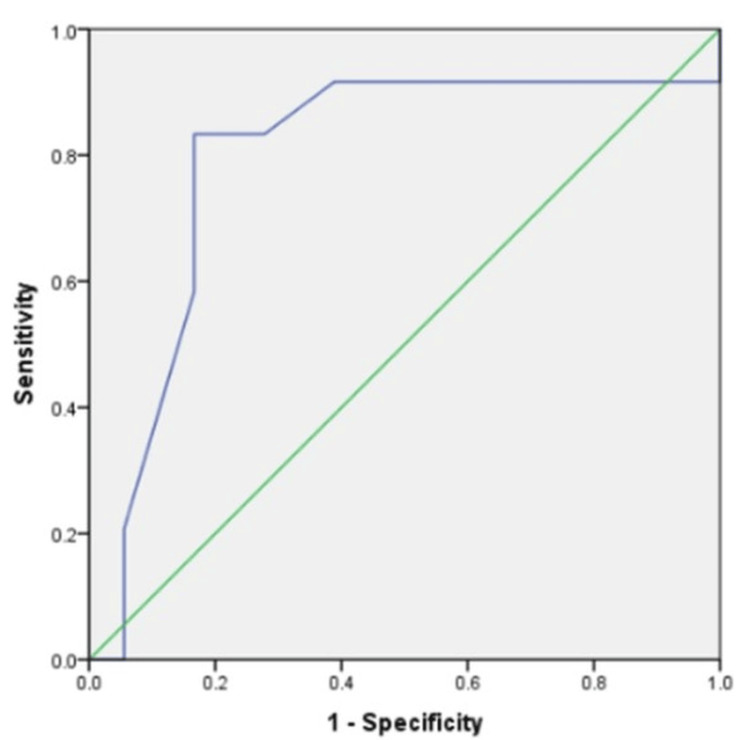
Receiver operator characteristics curve (ROC) showing the optimum cutoff ADC value for DWI. ADC: Apparent diffusion coefficient; DWI: diffusion-weighted imaging.

On a visual assessment, diffusion restriction (high signal intensity on DWI showing low intensity on corresponding ADC maps) was demonstrated by 66.67% (n=18) benign and 91.6% (N=33) malignant lesions. The mean ADC value for benign lesions was 1.27 x 10^3^ +/- 0.2 mm^2^/s and for malignant lesions was 1.08 x 10^3^ +/- 0.2 mm^2^/s. The optimum cutoff value found was 1.05 x 10^3^ mm^2^/s, which was statistically significant (the P value = 0.001). The area under the curve was 0.79 with a 95% confidence interval (Figure 4).

The sensitivity and specificity of ADC values were 83.3% each. The PPV, NPV, and accuracy were 85.7%, 78.5%, and 82.5%, respectively. Compared to individual MRI sequences DCE-MRI and DWI, the combined DCE-MRI with DWI showed a sensitivity equal to DWI imaging (83.3%) and a high specificity and accuracy of 94.4% and 88.1%, respectively. The PPV and NPV were 96.7% and 81.3%, respectively (Table 5).

**Table 5:** Comparative analysis of the statistical parameters of USG and MRI sequences for characterization of suspicious breast lesions in our study. USG: ultrasonography; DCE-MRI: dynamic contrast-enhanced MRI; DWI: diffusion-weighted imaging; ADC: apparent diffusion coefficient.

Study	Sensitivity (%)	Specificity (%)	PPV (%)	NPV (%)	Accuracy (%)
USG	91.7 %	63%	76.7%	85%	79.4%
DCE-MRI (kinetic curve analysis)	95.8%	78.5%	82.1%	85.7%	85.7%
DWI and ADC	83.3%	83.3%	85.7%	78.5%	82.5%
DWI+DCE-MRI	83.3%	94.4%	96.7%	81.3%	88.1%

**Figure 5:**
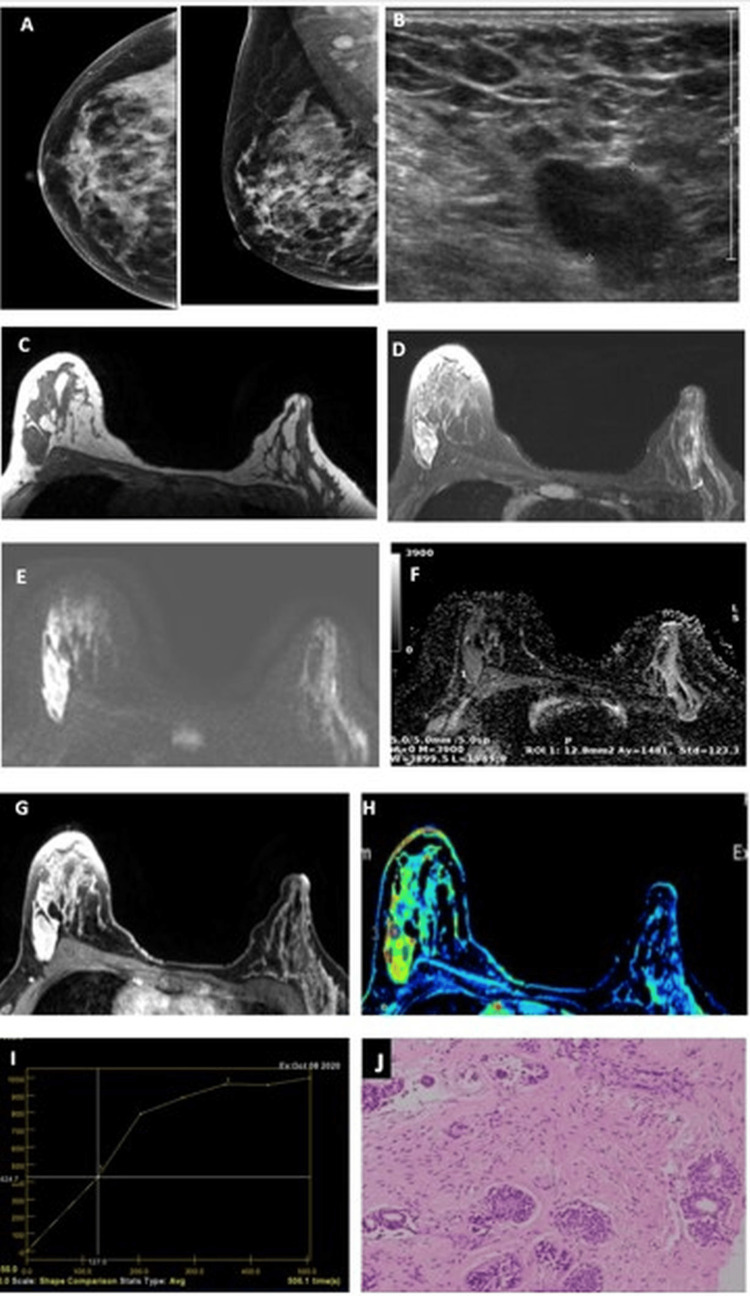
Fibrocystic disease of the breast. A 38-year-old female with a palpable lump in the right breast and a family history of breast cancer showed ACR BI-RADS type C breast composition on mammography (A) and focal asymmetry in the right upper outer quadrant. A USG (B) of the right breast showed an oval hypoechoic lesion with a circumscribed margin, categorized as likely benign (BI-RADS 3). An MRI of the right breast showed T1 (C) hypointense and T2 fat-suppressed (D) hyperintense oval lesion with a circumscribed margin; with diffusion restriction (E, F). There was a homogenous contrast enhancement in the dynamic VIBRANT sequence (G) and increased perfusion in the positive enhancement integral image (H). A kinetic curve analysis (I) showed a type I pattern categorized as a likely benign lesion. The histopathological (J) section (H&E stained: 100 X magnification) revealed dilated ducts lined by ductal epithelial cells containing secretions suggestive of fibrocystic disease. ACR BI-RADS: American College of Radiology Breast Imaging Reporting and Data System; USG: Ultrasonography; H&E: Hematoxylin and eosin

The ADC map generated from the DWI (b=800 mm^2^/s) showed higher values for fibrocystic disease (Figure 5) with increased vascularity on the positive enhancement integral image and type I kinetic curve.

**Figure 6:**
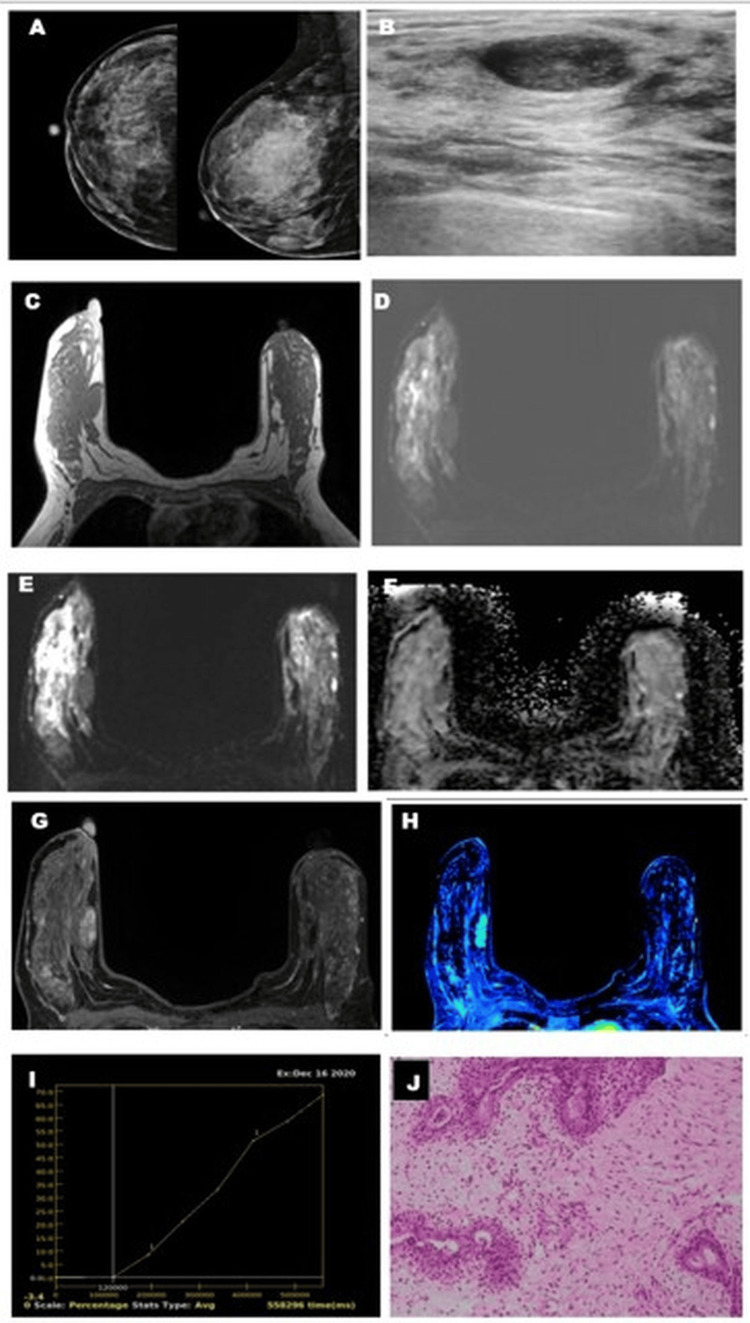
Fibroadenoma with type I kinetic curve on DCE-MRI. A 45-year-old female patient who presented with a vaguely palpable right breast lump with a family history of breast cancer showed ACR BI-RADS type D breast composition on mammography (A) without appreciable mass. USG (B) of the breast showed an oval hypoechoic lesion with a circumscribed margin, categorized as "likely benign." MRI imaging of the right breast showed a T1(C) hypointense lesion. The lesion appears hypointense on the T2 fat-suppressed image (D) with no restriction on DWI (E) and ADC (F). The postcontrast dynamic VIBRANT image showed homogenous enhancement (G) with no significant vascularity on the positive enhancement integral image (H). The kinetic curve analysis (I) showed a type I pattern, which was categorized as likely benign. Histopathological section (H&E stain, 100X magnification) (J) showed proliferation of glands and stroma suggestive of fibroadenoma. DWI: Diffusion-Weighted Imaging; ADC: Apparent Diffusion Coefficient; ACR BI-RADS: American College of Radiology Breast Imaging Reporting and Data System; USG: Ultrasonography; MRI: Magnetic Resonance Imaging; H&E: Hematoxylin and Eosin

**Figure 7:**
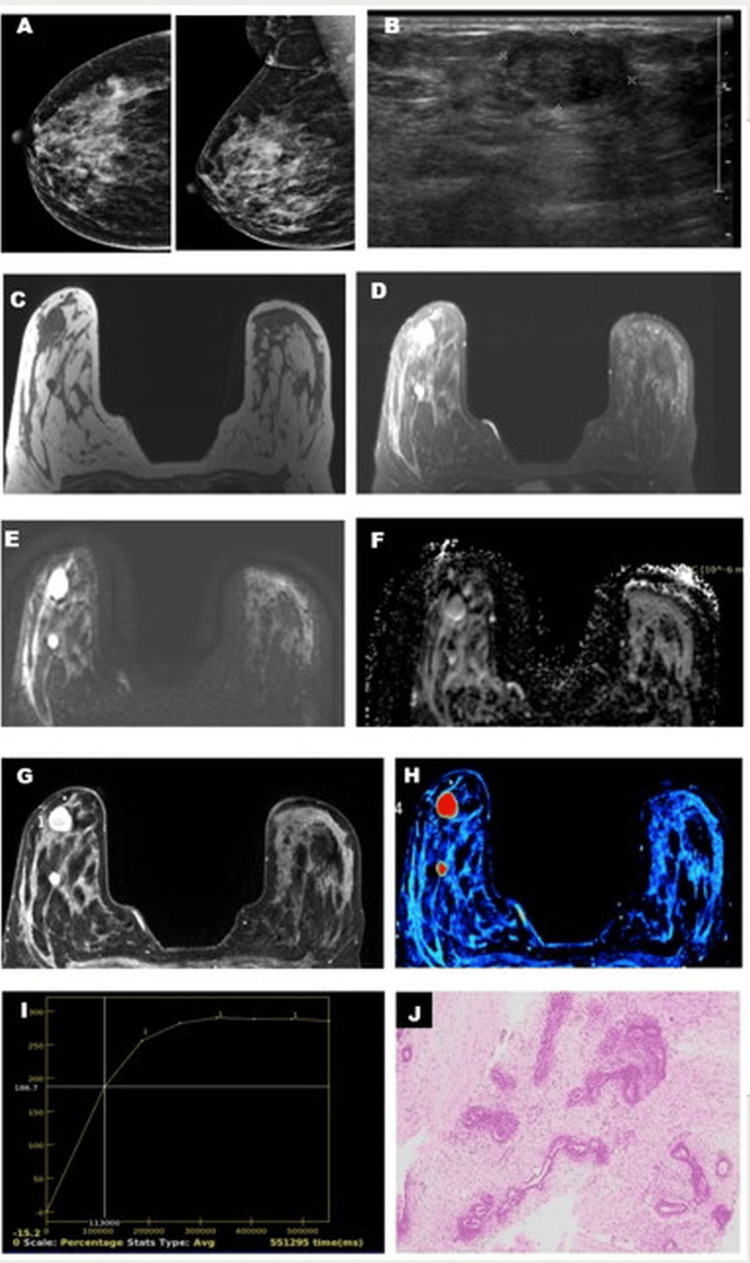
Fibroadenoma with type 2 kinetic curve on DCE-MRI. A 36-year-old female presented with a palpable lump in the right breast with ACR BI-RADS type C breast composition on mammography (A) without appreciable mass. A USG (B) of the right breast showed an oval hypoechoic lesion with a circumscribed margin and posterior enhancement categorized as likely benign. An MRI of the right breast showed two T1 (C) hypointense and T2 fat-suppressed (D) hyperintense oval lesions with circumscribed margins. Diffusion restriction (E) with the corresponding hypointensity on ADC was noted (F). There was a homogenous contrast enhancement of the lesion in the dynamic VIBRANT sequence (G), and positive enhancement integral image (H) showed increased perfusion. Kinetic curve analysis (I) showed a type 2 pattern. The histopathological (J) section (H&E stain, 100 X magnification) showed the proliferation of glands and stroma suggestive of fibroadenoma.

Fibroadenoma showed a higher ADC value with higher vascularity on the perfusion map and type I (Figure 6) or type II (Figure 7) kinetic curves.

**Figure 8:**
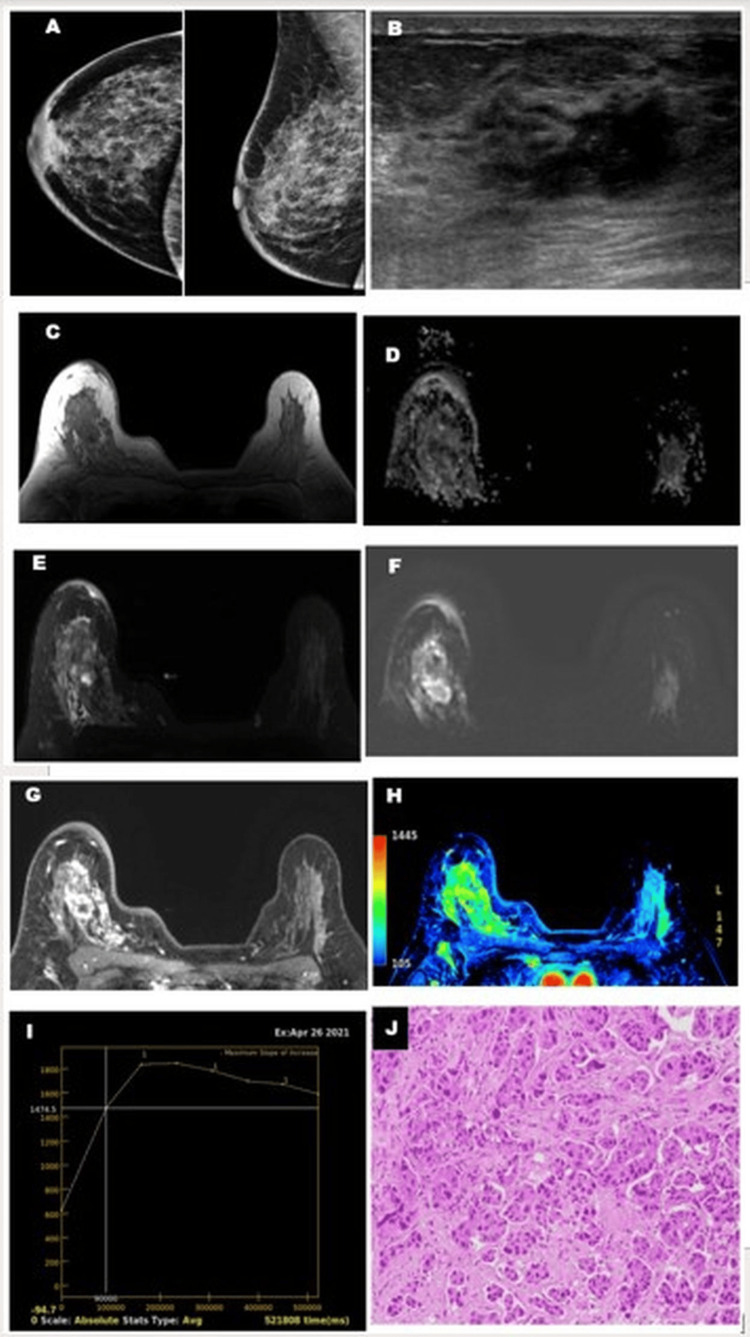
Invasive ductal carcinoma of the breast on USG and MRI. A 38-year-old female patient complained of pain in the right breast with ACR BI-RADS type C breast composition obscuring an underlying lesion in mammography (A), and no obvious mass was revealed. A USG (B) of the right breast showed an irregular hypoechoic lesion with a spiculated margin, categorized as likely malignant. An MRI of the right breast showed T1 hypointense(C) and T2 FS (D) heterogeneous lesions with irregular margin and diffusion restriction on DWI (E) and hypointensity on ADC imaging (F). There was heterogeneous contrast enhancement in the dynamic contrast study (G) with increased perfusion (H) on PEI within the lesion. Kinetic curve analysis (I) showed a type III pattern, categorized as likely malignant. Histopathological (J) section (H&E stain, 100X magnification) showed a cluster of small nests of malignant epithelial cells, polygonal in shape with a centrally placed nucleus and prominent nucleoli suggestive of invasive ductal carcinoma. ACR BI-RADS: American College of Radiology Breast Imaging Reporting and Data System; USG: Ultrasonography; MRI: Magnetic Resonance Imaging; T2 FS: T2 Fat-Suppressed; DWI: Diffusion-Weighted Imaging; PEI: Positive Enhancement Integral; H&E: Hematoxylin and Eosin

Invasive carcinoma (Figure 8) showed a lower ADC value with higher vascularity on the perfusion map and type III kinetic curves.

## Discussion

In our prospective observational study of the characterization of suspicious lesions in the dense breast by MRI compared to USG, we found that MRI is more accurate than USG in lesion characterization. Differentiating benign from malignant lesions can be done with high confidence by combining DCE-MRI with DWI. MRI can be recommended to characterize suspicious lesions in patients with dense breasts more accurately.

In the large screening mammography study by Checka et al., 74% of 7,007 participants with dense breast parenchyma were 40-49 years old [4]. Our study also had most patients (63.5%) in the age group of 30-50 years with a mean age of 40.

In our study, the commonest malignant pathology was invasive ductal carcinoma, and the most common benign pathology was fibroadenoma. It was similar to the results obtained by Li et al. in their breast lesion survey of 199,721 women, where invasive ductal carcinoma accounts for 56%, fibroadenoma for 20%, and invasive lobular carcinoma only 10% [12]. A study by Kim et al. showed that the irregular hypoechoic masses in the breast do not always indicate malignancies [13]. Many benign breast diseases such as fat necrosis, sclerosing adenosis, and inflammatory and granulomatous conditions present as irregular hypoechoic masses that can mimic carcinoma on USG. Our study also showed irregular shape in 45.8% of benign cases on USG.

In the Hong et al. study, 404 patients with breast lesions were analyzed and categorized according to USG BI-RADS. BI-RADS descriptors highly predictive of benign lesions were circumscribed margin (90%), and parallel orientation (78%); for malignant lesions, these were spiculated margin (86%), irregular shape (62%), and nonparallel orientation (69%) [14]. We had concordant results showing most of the benign lesions with circumscribed margins (66.6%) and parallel orientation (55.5%) and most of the malignant lesions showing spiculated margin (63.8%), irregular shape (63.8%), and nonparallel to the skin surface (72.2%).

The sensitivity, specificity, positive predictive value, negative predictive value, and accuracy of USG in our study were 91.7%, 66.7%, 78.6%, 85.7%, and 81%, respectively. The sensitivity and negative predictive value were high. At the same time, the specificity and diagnostic accuracy were relatively low compared to the study by Ghazala et al. and Tan et al. This can be attributed to interobserver variability as USG is operator-dependent [15,16].

The study by Hooley et al. showed that the false positive rate of USG is relatively higher in dense breast parenchyma [17]. Our study also showed similar results with a false positive rate of 38.8%. The sensitivity and specificity of USG in our study were 91.7 % and 66.7%, compared to Ghazala et al. showing sensitivity and specificity of 86.7% and 87.8%, respectively. The accuracy of USG in differentiating benign from malignant breast lesions in our study was almost similar to those of Ghazala et al., Tan et al., and Hashem et al. [15,16,18].

The current study considered both morphologic and kinetic parameters of DCE-MRI for MR BI-RADS categorization. Most patients showed mild background parenchymal enhancement without significant association with the lesion characterization. In their study, Hu et al. also showed no significant difference in background parenchymal enhancement between benign and malignant groups [19].

In our study, 83.3% of malignant masses showed an irregular shape, and 55.5% showed an irregular margin on MRI. Our findings are similar to the study with 969 participants by Mahoney et al., which suggested irregular shape, irregular margins, spiculated margins, and marked internal enhancement as strong predictors of malignancy [20].

The sensitivity of the DCE-MRI examination was 95.8%, while the specificity was 78.5% in our study. Our study yielded similar results as that of Singh et al. showing a sensitivity of 96% and specificity of 78.5% [21]. Another study by Ahluwalia et al. also showed similar results with a sensitivity of 95% and specificity of 70% [22]. Our results are comparable to those of a meta-analysis by Peters et al. to determine the diagnostic performance of MRI in patients with breast lesions showing a pooled sensitivity of 90% and specificity of 72% [23].

In our study, a type III washout curve was seen in 89% of malignant lesions. A type II plateau curve was seen in 11% of malignant lesions, and no malignant lesion showed a type I persistent curve. The type I curve was seen in 33.3%, and the type II curve in 66.6% of the benign lesions. No benign lesions showed a type III washout curve. Our findings concord with a study by Pinker-Domenig et al., who stated that the final diagnosis of malignancy was positively associated with a type III dynamic curve [24]. The sensitivity, specificity, and accuracy of DCE-MRI in suspicious dense breast lesion characterization were 97.2%, 70.3%, and 85.7%, respectively, which concord with studies by Ghazala et al., Hashem et al., and Kul et al. [15,18,25]. Pediconi et al. found in their study of a larger sample size (238) even higher values for the sensitivity, specificity, and accuracy of DCE-MRI in suspicious dense breast lesion characterization, which were 98.2%, 95.2%, and 96.9%, respectively [10].

The optimum cutoff from the ADC value in our study was 1.05x10-3mm 2/s. The study by Marini et al. showed a similar optimum ADC cutoff value of 1.1 x 10^3^ mm^2^/s [26]. The sensitivity and specificity of DWI to differentiate benign and malignant lesions were 83.3% each in our study. It was comparable to the results of Hashem et al., with a sensitivity of 73.2% and specificity of 83.7% [18]. In that study, the diagnostic accuracy of DCE was higher (85.6%) compared to DWI (78.9%). Our study also yielded a similar diagnostic accuracy of 85.7% for DCE-MRI and 83.3% for DWI. Kul et al. demonstrated an ADC cutoff value of 0.9 x 10^3 ^mm^2^/s with sensitivity and specificity of DWI in suspicious breast lesion characterization as 91.5% and 86.5%, respectively [25].

When we combined DCE-MRI with DWI (b=800 mm^2^/s), the specificity increased to 94.4% while only mildly affecting sensitivity (83.3%). These results are comparable with the meta-analysis from previous studies by Zhang et al., with pooled sensitivity and specificity of 91.6% and 85.5%, respectively [27]. Our results also agreed with the high values obtained by Kul et al., Tan et al., and Yadav et al. [25,28,29]. The accuracy of combined DWI and DCE-MRI in our study was 88% compared to 92.9% by Kul et al. [25]. Based on the present study, a combination of DCE-MRI with DWI is suggested to improve the specificity and diagnostic accuracy of lesion characterization of breast MRI.

The strengths of our study are that it was conducted in 3T MRI with a dedicated breast coil that provides high soft tissue contrast, spatial and temporal resolutions, and the use of multiparametric breast MRI for lesion characterization.

The limitations of our study are a small sample size and a single-center study.

## Conclusions

Thus, a combination of the advanced MRI sequences (i.e., DCE-MRI and DWI) better characterizes a lesion as benign or malignant in dense breast parenchyma than USG as they generate functional images. Because of its high diagnostic accuracy, MRI may be considered a better tool to solve the dilemma of dense breast lesions and diagnose breast cancer. USG can be used as a cost-effective modality in evaluating suspicious breast lesions in patients with MRI contraindications. Further multicentric studies with a larger sample size may help extrapolate our study results to the population.
